# Fatty Acid Metabolism Reprogramming in Advanced Prostate Cancer

**DOI:** 10.3390/metabo11110765

**Published:** 2021-11-09

**Authors:** Huan Xu, Yanbo Chen, Meng Gu, Chong Liu, Qi Chen, Ming Zhan, Zhong Wang

**Affiliations:** Department of Urology, Shanghai Ninth People’s Hospital, Shanghai 200011, China; 114023@sh9hospital.org.cn (Y.C.); 115039@sh9hospital.org.cn (M.G.); 119083@sh9hospital.org.cn (C.L.); chenq1392@sh9hospital.org.cn (Q.C.); zhanming@shsmu.edu.cn (M.Z.)

**Keywords:** neuroendocrine prostate cancer, castration-resistant prostate cancer, fatty acid, metabolic reprogramming

## Abstract

Prostate cancer (PCa) is a carcinoma in which fatty acids are abundant. Fatty acid metabolism is rewired during PCa development. Although PCa can be treated with hormone therapy, after prolonged treatment, castration-resistant prostate cancer can develop and can lead to increased mortality. Changes to fatty acid metabolism occur systemically and locally in prostate cancer patients, and understanding these changes may lead to individualized treatments, especially in advanced, castration-resistant prostate cancers. The fatty acid metabolic changes are not merely reflective of oncogenic activity, but in many cases, these represent a critical factor in cancer initiation and development. In this review, we analyzed the literature regarding systemic changes to fatty acid metabolism in PCa patients and how these changes relate to obesity, diet, circulating metabolites, and peri-prostatic adipose tissue. We also analyzed cellular fatty acid metabolism in prostate cancer, including fatty acid uptake, de novo lipogenesis, fatty acid elongation, and oxidation. This review broadens our view of fatty acid switches in PCa and presents potential candidates for PCa treatment and diagnosis.

## 1. Introduction

Prostate cancer (PCa) is the most commonly diagnosed malignancy in elderly males and the second leading cause of cancer-related deaths in western countries [1]. It accounts for 26% of all cancer diagnoses in males [1]. Because PCa grows relatively slowly, five-year survival rates tend to be high, approaching 99%, based on data from the Surveillance, Epidemiology, and End Results program. However, the occurrence of PCa still affects quality of life and decreases patient life spans. Unfortunately, typical treatments for PCa lack individualization. Though hormone therapy—including androgen deprivation therapy (ADT), which can be a standard systemic treatment for advanced PCa—eventually, the cancer becomes castration-resistant PCa (CRPC). Patients with CRPC have a median survival time of less than two years [2]. Radical prostatectomy is another effective treatment [3]. However, it is a challenge to treat metastases and invasive forms of CRPC [4]. The development of neuroendocrine prostate cancer (NEPC), a type of CRPC associated with small cell neuroendocrine cells, contributes to resistance to hormonal treatments, including treatment with enzalutamide, and ultimately leads to increased mortality [5]. There is still no effective therapy to combat NEPC in clinic work.

Altered lipid metabolism is known as a hallmark of PCa cells and thus represents a potential target for novel therapies [6]. In particular, altered activities of lipogenic enzymes have been connected to the genesis and development of PCa tumors [6], and additional insight into the process of reprogramming of fatty acid (FA) metabolism may yield effective therapeutic strategies.

In this review, we aim to summarize the reprogramming of FA metabolism in PCa, especially in advanced PCa. Both systemic FA metabolism in PCa patients and cellular FA metabolism in PCa cells are reviewed. FA metabolism may provide targets for the treatment or diagnosis of PCa.

## 2. Systemic FA Metabolism

### 2.1. Obesity and PCa

The incidence of PCa varies greatly around the world, with higher rates in Australia, New Zealand, North America, Western Europe, and Northern Europe [7]. One reason for the relatively high detection of PCa in these regions may be the availability of prostate-specific antigen (PSA) screening [8]. However, another reason may be the high rates of obesity in these areas [9]. Accordingly, recent meta-analyses have reported a positive association between obesity (one of the major risk factors for multiple human cancers) and PCa incidence [9,10], and there is compelling evidence linking obesity with the aggressiveness of PCa [11].

On the other hand, direct links between PCa incidence and obesity remain controversial. Some findings have reported a lack of association between obesity and PCa [12], and some have even reported a protective effect of obesity with respect to PCa initiation [13,14]. The differences in findings may be due to the nature of the prostate screening programs, as indicated by Emma H. et al. in 2013, in that obesity itself may lead to reduced PSA levels, and obese patients may be ineligible for biopsies that would provide definite diagnoses [15].

Though the relationship of PCa incidence to obesity remains somewhat controversial, multiple studies have shown that obesity correlates with higher risk for biochemical recurrence after radical prostatectomy and for PCa-specific mortality [16]. This effect may be due to obesity-related alterations of serum cytokines and some proliferative hormones, such as increased serum estrogen, insulin, insulin-like growth factor-1 and leptin, and reduced testosterone [17,18]. In addition, pathologic analyses also have revealed mechanisms that can explain the links between obesity and PCa advancement. In particular, it has been established that the uptake and storage of lipids in the form of lipid droplets by PCa cells play a vital role in the growth and development of PCa [19]; thus, obesity (and accompanying increased levels of serum lipids) contribute to the aggressiveness of PCa, though mechanisms explaining impacts of obesity on PCa initiation remain unclear.

Behavioral factors, including intake of food and nutrients, also strongly affect the incidence and progress of PCa. For PCa prevention, general nutritional guidelines, instead of individual foods, should be considered as recommendations. Increased vegetable and fruit consumption and decreased red meat and saturated fat intake have been suggested for the prevention of PCa [20]. With regard to specific nutrients, an investigation of potential protective roles of selenium and lycopene supplementation reported no significant effect of a 2 year therapeutic regimen on PCa [21]. Vitamin C supplements are also ineffective in preventing incident PCa, and vitamin E supplements may even increase risk of PCa, but more high-quality studies are needed [20]. Other specific nutrients that may impact PCa include ω3 and ω6 polyunsaturated fatty acids (PUFA), which cannot be synthesized de novo, and must be taken in from food [22]. Research has suggested that the consumption of the ω3 PUFA arachidonic acid promotes the development of PCa, while eicosapentanoic acid and docosahexanoic acid might have protective effects [23,24].

One recent report demonstrated that a Mediterranean diet pattern was associated with a lower risk of aggressive PCa; whereas, there was no relationship of PCa risk with Western or prudent dietary patterns [25]. However, a meta analysis in 2016 reported that a healthy diet pattern did not decrease PCa risk significantly (odds ratio (OR) = 0.96; 95% confidence interval (CI): 0.88–1.04). The Western diet pattern increased the incidence significantly (OR = 1.34; 95% CI: 1.08–1.65). There were also significant linear trends between the Western pattern (*p* = 0.011) and the carbohydrate pattern (*p* = 0.005) and PCa risk [25]. The relationships of various diet patterns to PCa are summarized in Table 1.

Multiple studies have observed that exercise and lifestyle may play important roles in the disease progression, mortality, and overall disease burden for PCa. Randomized controlled trials have demonstrated that more exercise is helpful for the decreased risk of cancer incidence and increased progression-free survival, PCa-specific survival, and overall survival. Exercise may also decrease progression to metastatic CRPC [38,39].

There are numerous potential reasons for the relationships between diet and exercise and PCa outcomes. First, the ratio of androgen to estrogen may be altered in different types of diet [40]. Secondly, as has been found in multiple epidemiologic studies, various foods may contain phytoestrogens (plant-derived xenoestrogens), intake of which may be related to a decreased risk of PCa [41]. A recently updated meta-analysis also confirmed this conclusion [42]. Thirdly, the gut microbiome is closely correlated with uptake of FA [43,44], and gut microbiome may influence cytokine activity throughout the system and in the prostate.

Though general dietary factors and precise nutrients may influence the PCa process, the status of the adipose tissue and the energy imbalance caused by diet, exercise, and lifestyle cannot be ignored. Moreover, visceral fat, which influences the enviroment of organs, is closely related to diet patterns [45], and a Mediterranean pattern, protein-enriched diet has been reported to decrease visceral fat [42]. In this way, food patterns may also influence the periprostatic adipose tissue (PPAT), which may contribute to PCa disease processes. There might also be biases in the analysis of life pattern and PCa; for example, this might be because family income might be different in individuals with different life patterns, which might contribute to the PCa screening and treatment.

### 2.2. Serum Lipid Metabolites in PCa Patients

**Figure 1:**
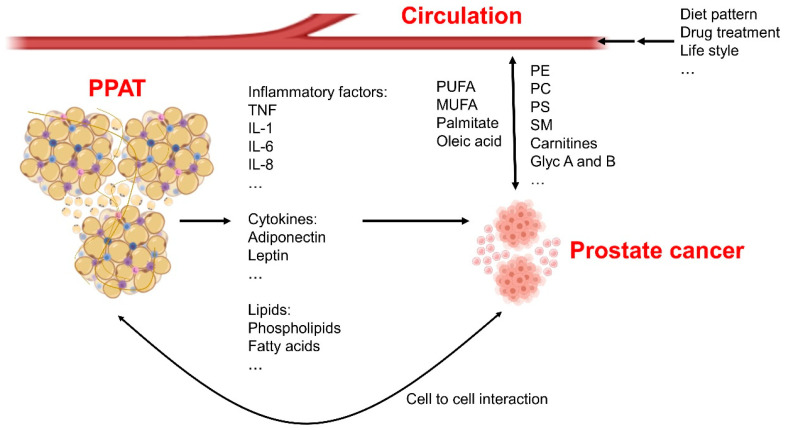
Systemic fatty acid metabolism changes in prostate cancer patients. The periprostatic adipose tissue (PPAT) is able to secrete inflammatory factors, cytokines, and bioactive lipids to the micro-environment of the prostate to affect growth and development of cancerous tissues. Cell-to-cell interactions also exist between adipocytes and prostate cancer cells, and these interactions contribute to cancer development. Circulating metabolites are changed because of diet patterns or lifestyles of individuals, these factors also affect cancer cells’ biology. Moreover, prostate cancer cells themselves can also secrete metabolites into the surrounding environment. Phosphatidylcholine—PC; sphingomyelin—SM; phosphatidylethanolamine—PE; phosphatidylserine—PS; poly-unsaturated fatty acid—PUFA; mono-unsaturated fatty acid—MUFA; interleukin—IL; tumor necrosis factor—TNF.

Peripheral blood-based liquid biopsies are of great importance for cancer diagnoses, as they detect cytokines, metabolites, and other circulating factors [46]. More importantly, the sampling is non-invasive (as compared with collecting tissue). However, the biggest drawback of serum tests is that they reflect the whole body, instead of the just the cancer. Analysis is needed to make this process more acurate for cancer detection. Though there are limits in liquid biopsy, they can reflect the status of the entire body and can have effects on the cells through circulation (Figure 1). Liquid biopsies can also provide evidence for better understanding of diseases [47]. Metabolic profiling is among the most widely used of liquid tests, and it plays significant roles in cancer diagnoses. The importance of metabolic profiling lies in the fact that metabolic reprogramming, which is frequently effected by oncogenes, is one of the most significant changes in cancers. Conversely, metabolites produced or regulated by tumors and the whole-body condition can also cause changes to gene expression epigenetically [48,49]. In recent years, improvements to mass spectrometry technologies and nuclear magnetic resonance have advanced the information obtainable through such tests for cancer detection [50,51,52]. For example, the use of nanoflow liquid chromatography–mass spectrometry (nano LC–MS) contributes to the detection of glycolipids, which can be used in clinical studies; thus, investigation of serum metabolites can be employed as a novel approach to gain further insight into cancer diagnoses and potential therapeutic targets.

Serum metabolic profiling panels have been reported to exhibit good diagnostic performance for the early detection of hepatocellular carcinoma from at-risk populations [53]. Similarly, changes to circulating metabolites in PCa have also been reported [47]. We have determined that a lipid metabolite biomarker panel (MET) exhibits good diagnostic performance for PCa detection. Lipids in this panel include N,N-dimethyl-phosphatidylethanolamine (18:0/18:2), phosphatidylcholine (16:0/20:2), phosphatidylserine (15:0/18:2), sphingomyelin (d16:0/24:1), and carnitine (C14:0). The diagnostic performance of this MET panel is particularly good in PCa patients with PSA levels of less than 20 ng/mL [47]. However, no correlation of the MET panel with Gleason scores was observed in PCa patients [47].

Moreover, using triple quadrupole liquid chromatography electrospray ionization tandem mass spectrometry, Chen et al. also performed a lipidomic profiling from 30 patients with PCa, 38 patients with benign prostatic hyperplasia (BPH), and 46 healthy male controls—the profiles indicated that the identified plasma lipid biomarkers have potential for the diagnosis of PCa [54]. Pathway analyses have also revealed a statistically significant association between lipids and PCa-specific death, and sterol or steroid metabolites showed the strongest chemical subclass association [55]. Moreover, carnitine-FA also plays important roles in the PCa detection. As is reported, acylcarnitines can separate PCa from benign prostatic hyperplasia in a patient derived serum study [56]. Carnitines attached to FAs and are related to FA oxididation. The specific carnitine levels (such as acyl carnitines) were reported to be associated with metastatic lethal PCa [57]. Thus, data from multiple research groups have demonstrated changes to circulating lipid levels in PCa patients. Lipid metabolites, sterols, steroids, and phospholipids in the serum or plasma might be good candidates for PCa detection, and findings correlating serum metabolomics to PCa may potentially be used for the development of new therapies for PCa [58].

### 2.3. Periprostatic Adipose Tissue (PPAT)

Obesity is related to PCa aggression and to the status of PPAT. The adipocytokines secreted by adipose tissues have multiple functions; however, their functions, with regard to the prostate gland, are partly due to the hormone receptors on the prostate epithelial cell directly and to the systemic metabolism indirectly. The mutual interactions that lead to specific functions are complicated and neuron net-like. Visceral fat, which surrounds organs, is different from subcutaneous fat, in that the visceral fat produces many cytokines and proinflammatory factors, which tend to induce an unhealthy enviroment for organs. Subcutaneous fat, on the other hand, is more of an energy and heat producer. As is reported in ovarian cancer, a majority of tumor cells can be transferred or located to the omentum, which contains substantive amounts of visceral adipose tissue [59]. Adipocytes also provide energy and adipocyte factors that encourage rapid tumor growth, causing the development of cancer and metastasis [59]. All of these factors indicate that the adipose tissue around the tumor can accelerate the cancer development. The prostate gland has an intimate physical relationship with visceral adipose tissue in that it tends to have a capsular-like structure that is surrounded by adipose, which makes adipocytes an important component of the organ’s environment. PCa often happens in the peripherla zone of prostate. The PCa cells tend to invade through the capsule infiltrating the PPAT. Thus, the adipokines and direct cell–cell contacts may influence the phenotypic behavior of cancer cells.

A clinical study has identified a relationship of the PPAT to the development and invasion of PCa. This study, by van Roermund et al. [60], utilized computerized tomography to identify an association between the area and density of PPAT and high-risk prostate cancer with PSA > 20 ng/mL, Gleason score ≥ 8, or stage ≥ T3. This study was designed to use transrectal ultrasonography to determine if the amount of PPAT is a risk factor for the incidence and aggressiveness of PCa, and it found that the amount of PPAT is a predictor of PCa and high-grade PCa at biopsy [61]. Iordanescu et al. [62] similarly used magnetic resonance (MR) analysis to measure the fatty acid composition of PPAT and found that fatty acid composition is altered in the PPAT of patients with aggressive PCa. The studies about PPAT and PCa are summarized in Table 2.

Though some hold the view that there is no correlation between PPAT density and PCa progression [64], most publications have reported that PPAT thickness or volume, as well as density, were independent predictors of PCa and high-grade PCa [60,61,68,69,70,71,72]. In addition to use in diagnosis, a study from Sumitomo et al. in 2010 [73] focused on the effect of PPAT on perioperative outcomes of high-intensity focused ultrasound (HIFU). HIFU was used to treat PCa, and the researchers evaluated whether obesity affected clinical outcomes. It was concluded that the thickness of the anterior perirectal fat tissue was one of the causative factors for poor clinical outcomes. Moreover, this relationship might help to explain the effect of obestity on PCa development and invasion. Indeed, the PPAT not only contributes to the HIFU outcomes, but also to the poor outcomes of radical prostatectomy. The presence of this fat pad may obstruct the surgeon’s view of the field of operation, and it may enhance adhesion to the surrounding tissues, via the action of cytokines secreted by adipocytes. Angiogenesis can also be accelerated by thick PPAT, elevating the surgical challenges and leading to poor post-operative outcomes [74,75].

Adipocytes (the mesenchymal stromal cells) interact with numerous different kinds of cells. Mammary adipocytes have been reported to significantly enhance casein and lipid accumulation within the mammary epithelial organoids [76]. Moreover, it has been shown that 3T3-L1 adipocytes stimulated the growth of SP1 cells, which represent murine mammary carcinomas, by secreting hepatocyte growth factor (HGF) [77,78]. Similar results also were reported in skin and cutaneous carcinoma cells in which the differentiation was promoted upon coculture with subcutaneous adipocytes [79,80]. As for the prostate, studies in cell culture suggest that cocultured adipocytes modulate the growth, morphology, and cytokine expression of PC3, a bone-metastatic prostate carcinoma cell line in a three-dimensional collagen gel matrix [81]. Angiogenesis is another vital progress that is induced by adipocytes, in part through the increase of the noted growth factors. However, there are also opposing effects from adipose tissue. As has been reported by our group, adiponectin defciency contributes to the development and progression of BPH as well as the growth of PCa cells [82,83].

The changes of the adipose tissues in obesity, including changes to the brown and white adipose tissues, is significant. Obesity also affects the biological characteristics of adipocyte in visceral fat. Obesity intensifies the tumor growth and development, and this provides a mechanistic hypothesis for the worse prognoses in obese PCa patients [84]. Adipose tissue is a metabolic organ producing hormones and cytokines that play multiple roles in the biology of PCa. The adipocytokines secreted in serum, in particular, tend to influence the progression of PCa. The adipocyte can secrete numerous hormones, including tumor necrosis factor, interleukin-6, leptin, ghrelin, and adiponectin [85]. Moreover, not only the hormones and adipocytokines but also the exosomes produced or stimulated from adipose tissue take part in the progression of cancer [86,87]. The enviroment around the prostate cells is partly constituted by adipocyte and the adipocyte-prostate cell interaction cannot be ignored.

PPAT also contains immune cells that have effects on the development of PCa. The lipid metabolism in the peripheral cells is also of importance for multiple cancers. As has been demonstrated by Kumagai et al., FAs provide an advantage for the function of Treg cells, which are able to present immunosuppressive functions within the environment of tumor cells [88]. In PCa, we also have previously reported that blocking N-cadherin or downregulating interleukin-8 is able to attenuate the immunosuppressive function that is caused by Treg cells. This might contribute to the PD-1 therapy resistance in advanced PCa [89]; thus, the metabolism in PPAT presents as a potential candidate to improve the target therapy sensitivity in PCa (Figure 1).

## 3. Cellular FA Metabolism

### 3.1. FA Concentration and Uptake in Prostate Cancer

FA concentrations are significantly upregulated in PCa tissues. This can be confirmed by magnetic resonance imaging (MRI). The most widely used technique is three-dimensional chemical shift imaging, and this technique has been used to demonstrate that lipids are relatively abundant in PCa tissues [90,91,92,93]. PCa tissues are not sensitive to analysis with ^18^F-glucose-positron emission tomography or computed tomography because of the limited glucose uptake and level of glycolysis. Glycolysis does increase with PCa development, and this technique becomes more sensitive in advanced PCa tissue. However, as lipid concentrations remain high in PCa tissues, other techniques using fatty acids are better candidates for detection and diagnosis [94,95]. The high level of FA may be caused by elevated FA uptake or by the upregulation of de novo lipogenesis. Though some short-chain FAs can be transported directly through the membrane, it is now generally recognized that FAs cross the cell membrane via a protein-mediated mechanism [96]. Researchers have also noted that a FA transporter (fatty acid translocase (CD36)) mediated metabolic changes and correlated with aggressiveness of PCa. In cell culture experiments, silencing CD36 in human PCa cells reduced FA uptake and cell proliferation. Deleting CD36 reduced fatty acid uptake and the abundance of oncogenic signaling in a mouse model of PTEN^−/−^ PCa [97].

Though there is close relationship between FA uptake and PCa progression, different types of FA have individual effects. The increased uptake of oleic acid and palmitic acid tend to increase cell proliferation, for instance [98]. However, excess palmitate causes oxygen stress, leading to apoptosis [99], and this effect can be prevented by pre-treatment with oleate or through triacylglycerol synthesis mediated by diacylglycerol O-acyltransferase 1 (DGAT-1), DGAT-1 is a gene involved in triglyceride synthesis. Accordingly, a DGAT1 inhibitor reduced the lipid droplet number and reduced the growth and development of PCa [19]. It is also reported that DGAT-1 has a protective role of DGAT1 for bone health [100]. The triglyceride metabolic process was shown to be elevated in invasive PCa cells. Adipose triglyceride lipase is important for the formation of DG and knock down of this protein reduced the rate of triglyceride hydrolysis and increased triglyceride levels in PCa cells [101].

As is reported for PC3 cells, which is a small cell PCa cell line, docosahexaenoic acid and eicosapentanoic acid show inhibitory effects on the uptake of phosphatidic acid and arachidonic acid [102]. In our previous study, we did not find that NEPC cells benefitted from the uptake of palmitate and oleic acid while arachidonic acid, a kind of PUFA, contributed to the activation of the AKT–mTOR pathway, inducing the neuroendocrine switch and enzalutamide resistance [24].

Thus, the concentration of various fatty acids might indicate the development of PCa and is a potential candidate target to overcome the drug resistance and NE differentiation.

### 3.2. De Novo Lipogenesis

To use FA as an energy source, normal prostatic cells rely mostly on diet-derived, circulating lipids (Figure 1). On the other hand, a study from the laboratory of Giorgia Zadra [103] suggests that PCa is marked by increasing rates of de novo FA synthesis. The key enzyme for this process is fatty acid synthase (FASN), which catalyzes the synthesis of palmitate from malonyl-CoA and acetyl-CoA, using metabolites that originate mainly from glucose or glutamine. Palmitate generation is followed by desaturation and elongation for the production of more types of FA. In many types of cancers, FASN is overexpressed and increased as the cancer develops. According to our studies of microarrays from prostate cancer patients, FASN is upregulated in PCa tissues and is increased with the elevation of Gleason’s score and clinical stages [104].

As reported by Richard Flavin, Giorgia Zadra, and Massimo Loda [105], natural sense and pharmacological inhibition experiments have shown the importance of FASN on proliferation and survival in multiple cancer cell lines. The involvement of the androgen receptor (AR) in the expression of FASN is likely. In prostate cancer, AR is critical for initiation and development. As a transcriptional factor, AR has been reported to activate sterol regulatory element-binding proteins (SREBPs), which play a central role in FA metabolism, especially in FASN expression. These connections can also explain why FASN and SREBPs are significantly increased in PCa tissues and cells, especially in metastatic CRPC cases [103,105].

In CRPC, there are many mechanisms leading to drug resistance, including AR amplification and hypersensitivity, AR mutations, androgen-independent AR activation, and intra-tumoral androgen production [106]. Among these, the AR pathway and the generation of the V7 splice variant (AR-V7) play important roles [107]. AR-V7 lacks the C-terminal ligand-binding domain of full-length AR (AR-FL), so this version of the receptor cannot be inhibited by androgen deprivation therapy because the receptor can be activated without ligands. It drives the growth of mCRPC cells’ escape from androgen deprivation therapy. AR-V7 mRNA and protein are up-regulated in PCa bone metastases, and overexpression was found in 39% of bone metastases. This variant was found to be consistently co-expressed with FASN [107] and associated with a decrease in overall survival [108] and resistance to either enzalutamide or abiraterone treatment, or both [109]. A FASN inhibitor, IPI-9119, was reported to reduce the growth of AR-V7-driven CRPC, both in xenograft models and human mCRPC-derived organoids. FASN inhibition can also elevate the enzalutamide sensitivity in CRPC cells [103]. These results suggest that FASN and AR are potential targets for the metabolic treatment of CRPC.

The upregulation of FA generation contributes to the architecture of the cellular membrane [110]. Moreover, it also contributes to the enhancement of cell signaling pathways, including the activation of the AKT–mTOR pathway and epigenetic regulation of k-RAS and WNT-1 [111]. It can also regulate endoplasmic reticulum function and resistance to genotoxic insults [112,113,114]. In addition, SREBPs are major downstream targets of the mTOR pathway, as evidenced by increased lipogenesis in response to mTOR activation, which is also consistent with its effect on hormone therapy resistance [115,116]. Chen et al. identified the hyperactivation of an aberrant SREBP promoted lipogenic program by MAPK reactivation. This program leads to a distinctive lipidomic profile as key characteristic features of PCa in which both *PML* and *PTEN* have been deleted [26]. More importantly, SREBP is a key factor in the regulating of tumor growth and distant metastasis in PCa, which can be regulated by AR [26,117]. Notably, loss of both *TP53* and *RB1* is one of the most important drivers of NE differentiation-induced hormone therapy resistance and lineage switching. The *RB1* gene can also interact with SREBP, which suppresses binding with target genes. Thus, in NPEC cells in which the retinoblastoma 1 gene has been deleted, SREBP has the potential to be upregulated, leading to the enhancement of de novo lipogenesis [118]. Specific *TP53* mutations can also interact with SREBP to increase its activation [118]. However, it remains unclear whether *TP53* loss affects cell development, and further studies are needed in this respect. ATP citrate lyase is also important in the de novo lipogenesis process. Though there have been no reports regarding prostate cancer, the effect of a feedback pathway has been observed involving ATP citrate lyase, AMP kinase, and AR attenuates tumor growth and the acquiring of cisplatin resistance in ovarian cancer [119]. As AR is a central regulator in PCa, this feedback pathway and its relationship with lipogenesis suggests additional mechanisms and targets in regard to PCa treatment.

### 3.3. Fatty Acid Elongation

**Figure 2:**
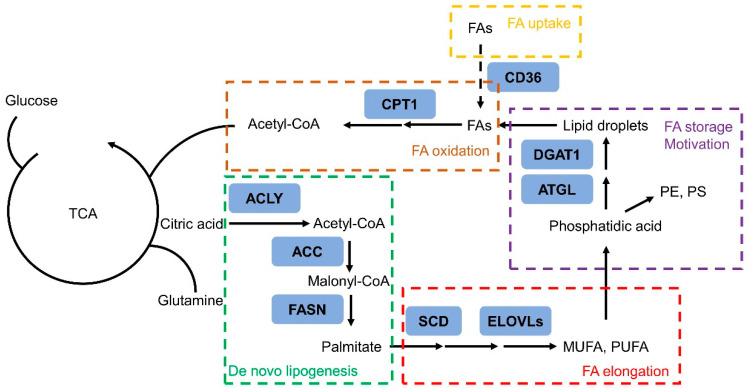
Schematic diagram of the rewiring of fatty acid metabolism in prostate cancer cells. Tricarboxylic acid cycle—TCA; mono-unsaturated fatty acid—MUFA; poly-unsaturated fatty acid—PUFA; fatty acid—FA; phosphatidylethanolamine—PE; phosphatidylserine—PS.

Fatty acid elongation is another critical pathway in FAs formation. Among the long-chain fatty acids, polyunsaturated fatty acids (PUFA) are fatty acids containing two or more double bonds, in which ω3 PUFA or ω6 PUFA refer to the position of the first double bond relative to the methyl end of the fatty acid (Figure 2). They have multiple functions which can influence the cellular fate. It has been reported that consumption of ω3 FAs reduced prostate tumor growth and increased survival, while ω6 FAs had the opposite effect [22,23]. When researchers introduced an ω3 desaturase, which converts ω6 FAs to ω3 FAs, into PTEN knockout mice, they identified a reducing effect on PCa growth [120]. Overall, as is generally understood, ω6 PUFA tend to accelerate inflammation, cancer cell proliferation, and metastasis, whereas, ω3 typically oppose these effects [22].

Members of the elongation of very long chain fatty acids (ELOVL) protein family are key enzymes involved in the FA elongation process. It has been reported that EVOLV7 is involved in PCa growth and negatively correlated with the survival of PCa patients. ELOVL7 is important for the synthesis of saturated very long chain fatty acids (SVLFAs) and their derivatives and may be a promising molecular target for the development of new therapeutic or preventive strategies for prostate cancers [121].

ELOVL5, as another ELOVL member, is the key enzyme for PUFA production. Work by Centenera et al. identified ELOVL5 as a pro-tumorigenic metabolic factor in PCa that is androgen-regulated and is critical for metastasis and PCa growth [122]. Intriguingly, according to our own research [24], FA elongation is enhanced after prolonged androgen deprivation therapy and in advanced PCa, including neuroendocrine PCa (NEPC). With additional arachidonic Acid (AA), one of the main PUFA, in the cell culture medium, prostate cancer cell lines present more enzalutamide resistance. When ELOVL5 is overexpressed, PCa cell lines shows elevated enzalutamide resistance, similar to the effect of adding extra AA to the growth medium. However, after the ELOVL5 downregulates, cells are more sensitive to hormone therapy. This effect is through the lipid raft-mTOR-AKT pathway, which is significant for CRPC treatment. ELOVL5 can also be significantly regulated by SREBP1-c and regulates the mTORC2-Akt-FOXO1 pathway by controlling hepatic cis-vaccenic acid synthesis in diet-induced obese mice [123]. In this way, the inhibition of long chain FA uptake might be a potential treatment for CRPC and to increase the hormone therapy sensitivity. Moreover, PUFAs play critical roles in ferroptosis regulation, which may be important for the protection of prostate cancer cells from damage from reactive oxygen species [124], which indicates that PUFA might contribute to the survival of NEPC and hormone therapy resistance through ferroptosis.

During the elongation process, desaturation is also needed. The key enzyme, stearoyl CoA desaturase (SCD), facilitates proliferation of prostate cancer cells through an AR dependent pathway [125,126]. An SCD1 inhibitor (BZ36) has been proven to repress the proliferation of LNCaP and C4-2 cells in vitro and in vivo through the phosphatidylinositol 3-kinase and AKT-dependent pathway [127,128]. Researchers have also studied long-chain acyl CoA synthetase (ACSL) enzymes in terms of their role in providing fatty acyl-CoAs, which are downstream metabolites of FA. ACSL1 was shown to regulate production of various lengths of acyl-CoAs in cancer cells. Expression levels of ACSL1 was elevated in PCa, contributing to the proliferation and migration of prostate cancer cells in vitro and in vivo [129].

### 3.4. Fatty Acid Oxidation

FA oxidation is typically only associated with energy harvesting (Figure 2). However, fatty acid reprogramming should also be placed in a different context: as a critical gatekeeper that is regulated by oncogenic signals to drive cancer growth and development. In PCa cells, FA oxidation is increased, and the key enzyme, carnitine palmitoyltransferase 1 (CPT1)—which catalyzes the transfer of long-chain FA into the mitochondria for further oxidation—is upregulated [130,131]. The dominant metabolic role of FA oxidation, rather than glycolysis, has the potential to fuel PCa growth and to be the basis for imaging-based diagnoses and targeted treatment of PCa [132]. Findings have also investigated that carnitine system could regulate the metabolic flexibility of cancer cells, which plays a fundamental role in switching between the glucose and FA metabolism. The carnitine is pivotal to tumor growth and survival [133]. MicroRNAs that targeted the carnitine system also affected tumorigenic properties, such as proliferation, migration, and invasion, in both PC3 (AR negative) and LNCaP (AR positive) cell lines [134]. Moreover, a recent study using matrix-assisted laser desorption ionization time-of-flight (MALDI-TOF) mass spectrometry imaging (MSI) also indicated the increased levels of carnitine shuttle in prostate cancer tissues [135].

Cellular FA oxidation produces NADPH, which scavenges reactive oxygen species to protect cells from oxidative stress and protect cells from oxidative stress [136]. FA oxidation has been shown to be a driver of cancer metastasis and to be important for activation of the FA binding protein 12-peroxisome proliferator-activated receptor γ pathway, which has a role in metastasis of PCa through modulation of the epithelial–mesenchymal transition process [137,138]. Moreover, PCa metastasis is enhanced by the delivery of FA to nuclear receptors by FA binding protein 5 (FABP5). This protein is not expressed in normal prostate but is highly upregulated in metastatic PCa. The pro-metastatic effects of FABP5 are through PPAR and estrogen-related receptor α pathways [139,140]. Thus, the use of FA, either for energy or for nuclear transportation, is a critical determinant of cellular fate.

However, in our previous research [24], we did not find that FA oxidation was enhanced in CRPC-NEPC cell lines. Instead, the source of oxidation mainly depends on the FA present in the medium. LNCaP/AR-shp53/shRB PCa cells—which are NE-like PCa cells—are less sensitive to FA depletion than LNCaP/AR cells are, which indicates that NE-like PCa cells may depend less on FA oxidation.

The significance of FA metabolism can be probed with etomoxir, the most widely used inhibitor of CPT1, and hence, block the carnitine shuttle. This inhibitor blocks the entry of FA into the mitochondria, where FA would be subject to further oxidation and energy production. It is a safe irreversible inhibitor, having been used in the treatment of heart failure [141]. When treated with etomoxir, the decreased proliferation level of NE-like PCa cells is less, when compared with PCa adenocarcinoma cells. This may be caused by the increased use of glutamine as fuel in NEPC cells [142], which is caused by the decreased expression of kidney-type glutaminase (KGA) and upregulation of glutaminase 1 in hormone therapy resistant and NEPC cells [142]. Studies from the lab of Jiaoti Huang demonstrated that NEPC cells become dependent upon glutamine but not on glucose, and that the splicing form switch is induced by androgen receptor [142]. All these factors might contribute to the lesser energy decrease after CPT1 inhibition in NEPC cells, compared with adenocarcinoma cells. Interestingly, CPT1C, which is expressed at a low level in cells and not the main form of CPT1 enzyme, is critical for the growth and development of small cell lung cancer. CPT1C is mainly expressed in neurons and can be induced by hypoxia and glucose deprivation [143]. In breast cancer, CPT1C overexpression increases rapamycin resistance and it might act in parallel to mTOR-enhanced glycolysis [144]. As CPT1C expression is closely related to the behavior of neuroendocrine cells and to the process of glycolysis, this enzyme might be a candidate for the development of NEPC-targeted therapies. Though it is not the main type for CPT1, it might still play important roles in the NEPC cell development or the NE differentiation. In a recent publication, the expression of the related enzyme CPT2 has also been reported to be significantly correlated with therapy resistance in PCa; thus, it might be a predictive marker [145,146]. Further studies are needed to take advantage of the connections between FA oxidation, NEPC differentiation, and therapy resistance.

## 4. Conclusions

FA metabolism reprogramming contributes to initiation and development in PCa. Both systemic and cellular FA metabolism is significantly rewired in PCa. FA metabolism pathways are potential candidates for diagnosis and treatment of prostate cancer. Especially in advanced prostate cancer, targets to FA metabolism tend to elevate therapy sensitivity and decrease the disease’s progression.

## Figures and Tables

**Table 1:** Diet patterns and prostate cancer (PCa).

Diet Pattern or Nutrients	Species (Human or Mice)	Major Findings	References
High fat diet	Human, mice	High fat diet fuels prostate cancer progression.	[26,27,28]
Dietary Approaches to Stop Hypertension (DASH)	Human	DASH diet, may reduce the odds of high aggressive prostate cancer. A weaker inverse association between DASH scores and prostate cancer aggressiveness was observed.	[29]
Mediterranean	Human	Higher diet quality, as represented by a Mediterranean-style diet, reduces the rate of highly aggressive prostate cancer.	[29,30,31]
Western dietary pattern	Human	Western dietary pattern increases prostate cancer risk.	[32,33,34]
Chinese Food Pagoda	Human	Not reported in PCa. However, it has shown that it is related to DNA methylation, histone modifications and non-coding RNA expression in cancer cells to attenuate tumor progression and prevent metastasis.	[35,36,37]

**Table 2:** Periprostatic adipose tissue (PPAT) and prostate cancer (PCa).

Species (Human, Mice, or Cell Line)	Study Design	Major Findings	References
Human	Clinic study	48% of PPAT is on prostate surface, 57-59% on the right and lateral surface, 44% and 36% along the anterior and posterior region.	[63]
Human	Clinic study	PPAT area and density were not associated with PCa aggressiveness.	[64]
Human	Clinic studies	Significant association between total PPAT area and density with high-risk, more aggressive and developed PCa.	[48,49,52,53,54,55,56]
Human	PPAT derived from patients	Elevated expression of IL-1 and IL-6 in PCa sample in comparison with normal prostate.	[65]
Human	PPAT derived from patients	Higher secretion of IL-6 from PPAT was observed in higher tumor grade.	[11]
Cell lines	PPAT derived from patients	PPAT derived factors increased migration of both PC3 and LNCaP cell lines, while PPAT had a strong proliferative effect on PC3 cell lines.	[66]
Cell lines	PPAT derived from patients	Conditioned media from PPAT obtained from patients with prostate cancer: integrin family cells surface interaction and homeostasis pathway were enriched pathways in tumor cells after cell medium culture.	[67]
